# When Thiamine Is Not the Answer: Sporadic Creutzfeldt-Jakob Disease Mimicking Wernicke’s Encephalopathy

**DOI:** 10.7759/cureus.105342

**Published:** 2026-03-16

**Authors:** Carlos Fagundo, Catherine Xu, Reyleen Loreto, Hazem Abugrara

**Affiliations:** 1 Internal Medicine, Baycare Health System, Tampa, USA; 2 Hospital Medicine, Baycare Health System, Tampa, USA

**Keywords:** alcohol use disorder (aud), creutzfeldt–jakob disease, encephalopathy, prion disease, rapidly progressive dementia

## Abstract

Creutzfeldt-Jakob disease (CJD) is a rare and rapidly progressive neurodegenerative disorder caused by the accumulation of misfolded prion proteins, resulting in neuronal damage. It can present with symptoms that mimic other neurological conditions, which can complicate early diagnosis. We report a case of a 58-year-old male who presented with cognitive decline, motor dysfunction, and behavioral changes following alcohol cessation, initially resembling Wernicke’s encephalopathy. As his condition progressed, he developed nonconvulsive status epilepticus, and subsequent diagnostic workup demonstrated cortical diffusion restriction on MRI together with a positive real-time quaking-induced conversion (RT-QuIC) assay and elevated cerebrospinal fluid (CSF) 14-3-3 protein levels, confirming the diagnosis of CJD. This report highlights the diagnostic difficulties associated with prion diseases, where early symptoms may overlap with more treatable neurological conditions, and underscores the importance of considering prion disease in cases of rapidly progressive dementia that do not respond to thiamine therapy.

## Introduction

Creutzfeldt-Jakob disease (CJD) is a rare and rapidly progressive prion disease that primarily affects the central nervous system, leading to severe neurodegeneration and death. CJD is classified into four main types: sporadic CJD (sCJD), genetic CJD (gCJD), iatrogenic CJD (iCJD), and variant CJD (vCJD). Sporadic CJD is the most common form, accounting for over 80% of cases, and typically occurs in individuals without a known genetic or environmental trigger [1]. It is caused by the accumulation of misfolded prion proteins (PrP^Sc^), which induce conformational changes in normal prion proteins (PrP^C^), resulting in widespread neuronal damage. The disease typically presents in the sixth to seventh decade of life and affects men and women equally, although some epidemiologic studies report a slightly higher prevalence among men aged 55 to 75 years [2,3].

The clinical presentation of Creutzfeldt-Jakob disease can be highly variable, often mimicking more common neurodegenerative or psychiatric disorders such as Alzheimer's disease, Lewy body dementia, or Wernicke’s encephalopathy, which complicates early diagnosis. Diagnostic challenges are further exacerbated by the absence of specific biomarkers in the early stages of the disease. Currently, no disease-modifying therapies are available, and management remains supportive and palliative. We discuss the case of a 58-year-old male who initially presented with symptoms similar to Wernicke’s encephalopathy following alcohol cessation, later developed nonconvulsive status epilepticus, and was ultimately diagnosed with CJD through clinical evaluation, neuroimaging, and cerebrospinal fluid (CSF) analysis.

## Case presentation

The patient was a 58-year-old male who presented in 2025 with a history of rheumatoid arthritis, benign prostatic hyperplasia, diverticulitis, and alcohol use. Approximately two months before admission, the patient had suddenly stopped drinking alcohol, after which he had started showing signs of inappropriate behavior and cognitive decline. The patient's spouse had noticed increasing difficulty with word-finding, memory impairment, gait instability, and an inability to carry out daily tasks. Importantly, he had once tried to buy two items from a store but returned home empty-handed, leading his spouse to seek medical care.

**Figure 1:**
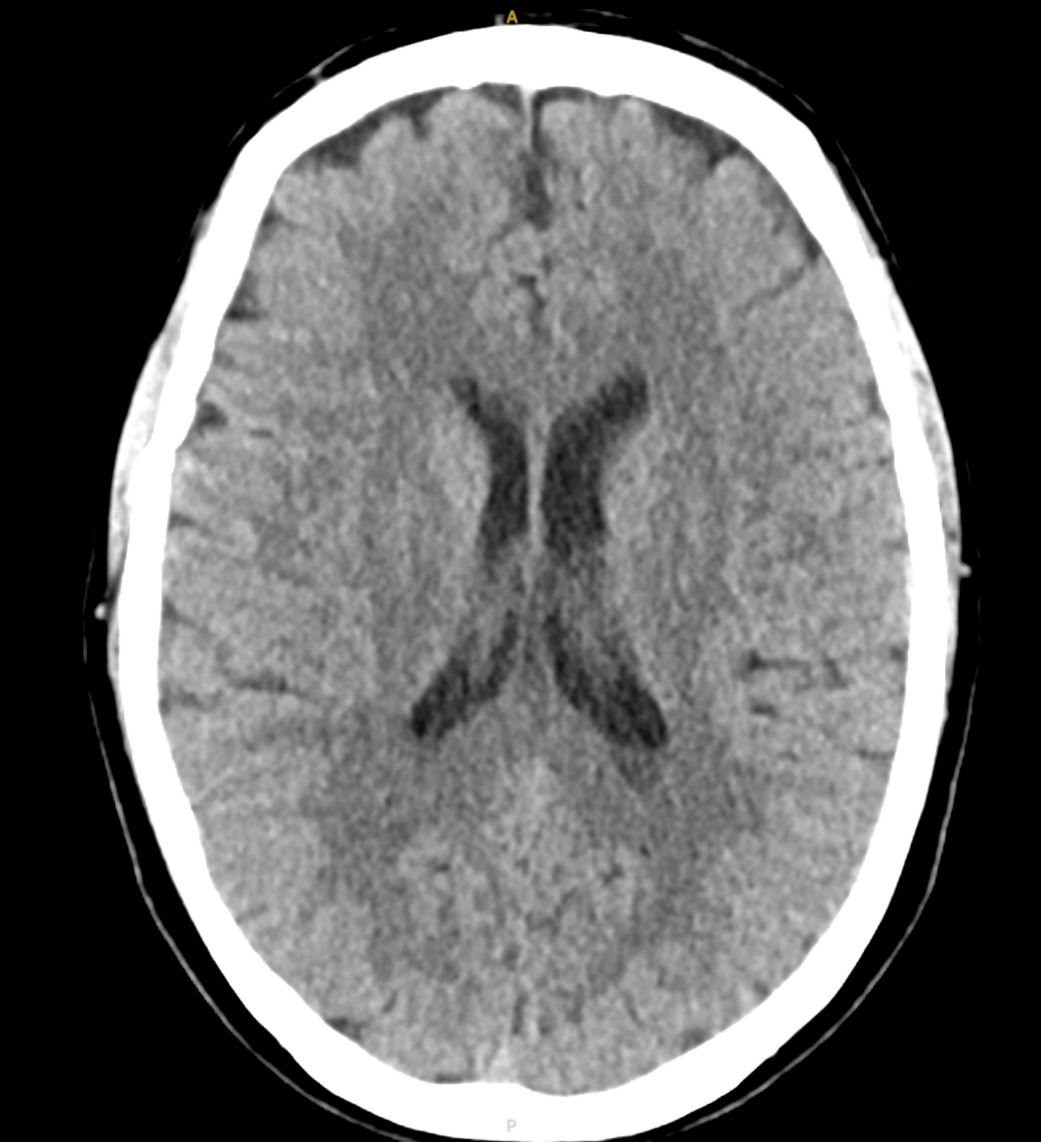
CT of the head without contrast There is no acute intracranial mass, mass effect, or midline shift. There is no evidence of intra- or extra-axial hemorrhage. The ventricles appear normal, and the sulci and gyri are appropriate for the patient’s age CT: computed tomography

On presentation to the emergency department, the patient’s vital signs were stable, except for mild tachycardia (heart rate: 101 beats per minute). Physical examination revealed no visual hallucinations, nystagmus, ophthalmoplegia, paresthesia, spasticity, or glossitis. His cognitive function was noted to be impaired, and he exhibited mild ataxia and cognitive slowing. Initial laboratory workup, including a complete blood count (CBC) and complete metabolic panel (CMP), showed no significant abnormalities. A urine toxicology screen was positive for cannabis, serum vitamin B12, and syphilis screening were within normal limits (Table 1). A non-contrast CT scan of the head revealed no acute intracranial abnormalities (Figure 1). The patient was admitted to the medical floor for further evaluation of his encephalopathy, and empiric intravenous thiamine was administered.

**Table 1:** Laboratory and CSF results CSF: cerebrospinal fluid; RT-QuIC: real-time quaking-induced conversion assay; RPR: rapid plasma reagin

Test	Patient value	Reference range	Interpretation
Urine toxicology screen	Positive for cannabis	Negative	Abnormal (positive)
Vitamin B12	639 pg/mL	200–900 pg/mL	Normal
Syphilis screening (RPR/treponemal)	Non-reactive	Non-reactive	Normal
CSF cell count	Normal	0–5 WBC/µL	Normal
CSF glucose	Normal	40–70 mg/dL	Normal
CSF protein	Normal	15–45 mg/dL	Normal
CSF 14-3-3 protein	61,713 AU/mL	173–1,999 AU/mL	High
CSF total tau protein	>20,000 pg/mL	<1,149 pg/mL	High
RT-QuIC assay	Positive	Negative	Positive

**Figure 2:**
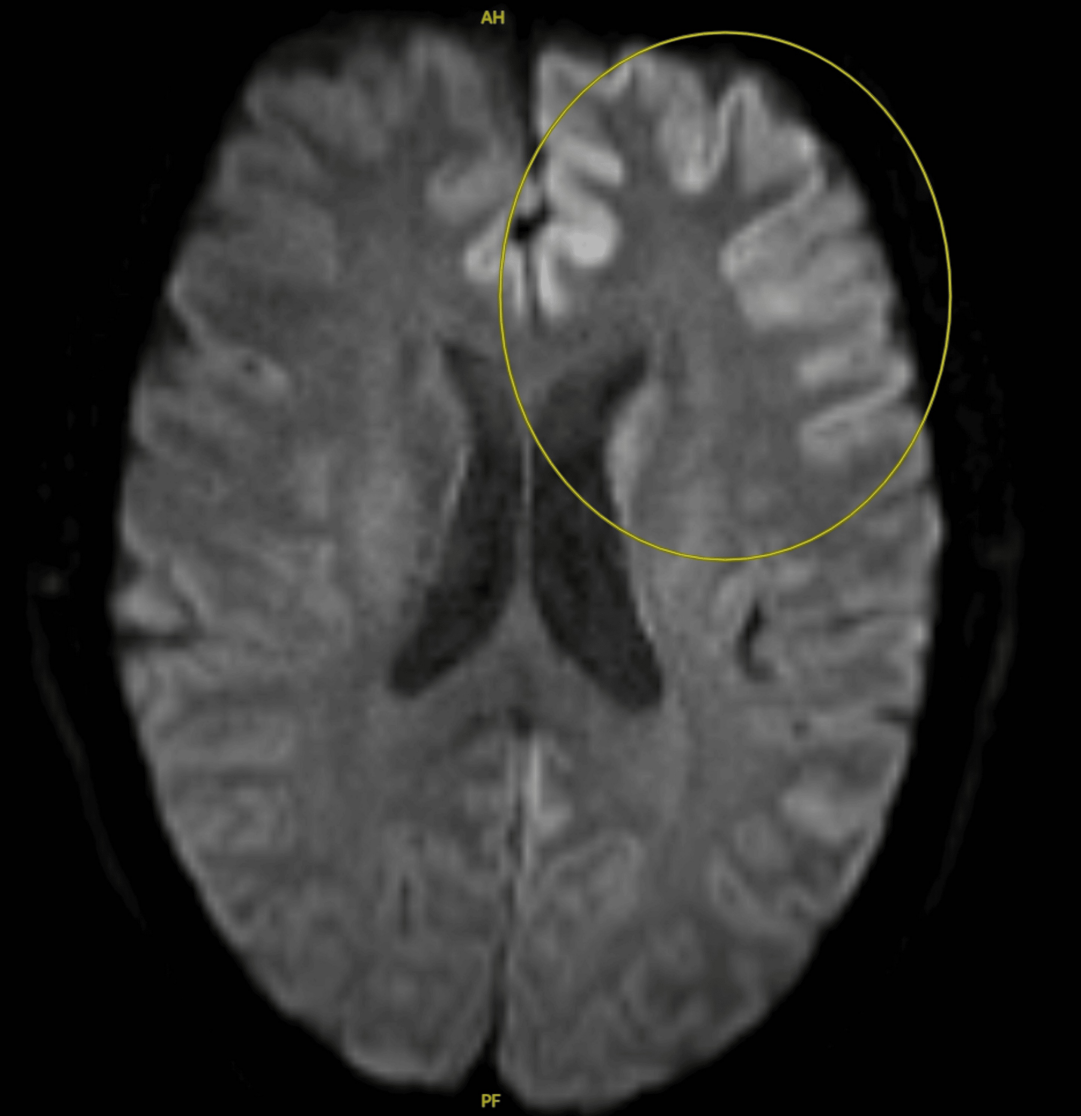
MRI without contrast The findings are notable for cortical diffusion restriction in the left frontoparietal lobes (circled in yellow) and, to a lesser extent, involving the right frontoparietal lobes MRI: magnetic resonance imaging

Further workup with MRI of the brain demonstrated asymmetric cortical diffusion restriction in the left frontoparietal lobes, bilateral insula and operculum, and, to a lesser extent, in the right parietal cortex, raising concern for prion disease (Figure 2). A neurologist was consulted, and a lumbar puncture was performed, revealing clear CSF with normal cell count, glucose, and protein levels; however, with significantly elevated levels of CSF 14-3-3 protein elevated tau protein, and a positive real-time quaking-induced conversion (RT-QuIC) assay suggestive of CJD (Table 1).

During hospitalization, the patient developed recurrent seizures, which advanced into nonconvulsive status epilepticus as confirmed by continuous electroencephalography (EEG) monitoring. The patient received a regimen of high-dose methylprednisolone (1 g IV daily for three days) and multiple antiepileptic drugs, including eslicarbazepine 1200 mg twice daily, phenytoin 200 mg every 12 hours, lacosamide 200 mg twice daily, and valproate sodium 500 mg four times daily. Despite escalating therapy, the patient continued to experience lateralized epileptiform discharges and worsening encephalopathy, requiring transfer to the neurointensive care unit for mechanical ventilation and closer monitoring.

Over the following weeks, the patient remained intubated and sedated. Repeat MRI showed persistent cortical ribboning and restricted diffusion, consistent with prion disease. A second neurological opinion was obtained and confirmed the diagnosis of sCJD, based on the characteristic clinical presentation, neuroimaging findings, and CSF biomarker profile. Given the poor prognosis and lack of meaningful neurological recovery, discussions regarding palliative care were held with the patient’s wife and family. The patient subsequently died following the transition to comfort-focused care.

## Discussion

CJD is a rare, rapidly progressive neurodegenerative disorder resulting from misfolded proteins that cause severe neuronal damage. While it is considered an infectious disease due to the ability of misfolded prion proteins (PrPˢᶜ) to induce similar misfolding in normal proteins (PrP^C^), it is not contagious in the conventional sense, as seen with bacterial or viral infections. Transmission of CJD occurs through exposure to contaminated tissue but cannot occur via casual contact [1]. This unique mode of transmission, combined with the disease's rarity and rapid progression, makes diagnosing and managing it particularly challenging. 

CJD arises from a mutation that alters the normal folding of the cellular PrPᶜ, converting it into its pathogenic form, PrPˢᶜ. The abnormal PrPˢᶜ induces misfolding of other native prion proteins, resulting in progressive accumulation within the central nervous system. Over time, these misfolded proteins aggregate into insoluble amyloid plaques, which are resistant to degradation and contribute to widespread neurodegeneration [2]. PrPˢᶜ aggregates are detrimental to neurons, resulting in spongiform degeneration. This degeneration is marked by a reduction in neuronal population and gliosis, all of which transpire without a notable inflammatory response [2,3,4].

Among the four types of CJD, sCJD accounts for approximately 85% of cases and develops without identifiable provoking factors, whether genetic or environmental. It most commonly occurs between the ages of 60 and 65 years [2,5]. gCJD is linked to a mutation in the PRNP gene on chromosome 20, which encodes the normal prion protein, PrP^C^. This condition is inherited in an autosomal dominant fashion and accounts for about 10-15% of all CJD cases. This condition typically manifests in individuals aged 40-50 years.

iCJD refers to prion infection transmitted through certain medical procedures, most commonly involving dura mater grafts or treatment with human-derived pituitary growth hormone. Diagnosis depends on identifying a history of potential exposure along with clinical manifestations that resemble those seen in sCJD. vCJD predominantly affects younger individuals, with an average age of onset of 27 years. Early in the disease, patients often present with prominent psychiatric symptoms, which progressively worsen as significant neurological decline occurs. This form of CJD is linked to bovine spongiform encephalopathy (BSE), also known as mad cow disease, and is characterized by the presence of florid plaques.

This case highlights the challenges in distinguishing CJD from other, more common conditions, especially in its early stages, when symptoms often overlap with those of other neurological and psychiatric disorders. The diagnosis of suspected CJD requires a multimodal approach. Brain MRI remains one of the most sensitive and specific diagnostic tools, particularly when using diffusion-weighted imaging (DWI) and FLAIR sequences. Characteristic findings include cortical ribboning and signal abnormalities in the basal ganglia, thalamus, or cortex. In our patient, an early MRI showing parietal and frontal cortical diffusion restriction with cortical ribboning was a key finding that prompted further cerebrospinal fluid evaluation for prion disease (Figure 2). These MRI features have been reported to have a sensitivity of 91% and a specificity of 97% for diagnosing CJD.

The clinical course of CJD is often marked by rapidly progressive dementia, myoclonus, ataxia, and psychiatric disturbances. This case illustrates the variability in clinical manifestations, as our patient developed progressive cognitive dysfunction, memory impairment, and gait instability, which could easily have been attributed to alcohol-related brain injury or nutritional deficiencies. However, the occurrence of nonconvulsive status epilepticus, along with elevated 14-3-3 protein and tau protein in the cerebrospinal fluid, was pivotal in distinguishing CJD from other conditions with similar symptoms. This combination indicates rapid neuronal injury consistent with a prion-mediated neurodegenerative process rather than a reversible metabolic or nutritional encephalopathy. Although these biomarkers are suggestive, they are not specific and may be present in other acute neurological disorders, making the diagnosis of CJD particularly challenging.

Patients with alcohol use disorder who present with cognitive decline, confusion, and gait instability are often evaluated for Wernicke’s encephalopathy, a condition linked to vitamin B1 deficiency. Wernicke’s encephalopathy typically responds to thiamine supplementation, and when patients fail to improve, alternative diagnoses like CJD should be considered. 

Among chronic neurodegenerative diseases, Alzheimer’s disease remains the leading cause of dementia in older adults and typically develops insidiously, with gradual memory impairment. It is pathologically defined by the presence of β-amyloid plaques and tau-related neurofibrillary tangles, resulting in progressive neuronal loss and atrophy [8]. In contrast, CJD progresses over a period of weeks to months and is often accompanied by myoclonus, ataxia, and visual disturbances [9]. Lewy body dementia presents with fluctuating cognition, visual hallucinations, and Parkinsonism [10], whereas frontotemporal dementia usually affects younger individuals and manifests with early behavioral or language changes [11].

Pathologically, these conditions differ: CJD is characterized by prion-mediated spongiform degeneration, Alzheimer’s disease by β-amyloid plaques and tau tangles, Lewy body dementia by α-synuclein deposits, and frontotemporal dementia by tau or TDP-43 inclusions. Diagnostic approaches also vary. CJD is supported by MRI findings such as cortical ribboning, cerebrospinal fluid biomarkers, RT-QuIC assays, and EEG abnormalities, whereas the other dementias are diagnosed primarily based on characteristic clinical features, structural imaging, and disease-specific biomarkers.

RT-QuIC has emerged as the most specific assay, detecting misfolded prion protein with high diagnostic accuracy. A positive RT-QuIC not only supports the diagnosis but also provides clarity for prognosis and timely transition to palliative care. Its incorporation into international diagnostic criteria underscores its central role, though access remains limited in some centers [3,6,12].

## Conclusions

This report highlights the complexity of diagnosing sCJD, particularly when early symptoms overlap with more common and potentially reversible conditions such as Wernicke’s encephalopathy. Our patient’s rapid neurologic decline, development of nonconvulsive status epilepticus, and confirmatory MRI and CSF findings ultimately established the diagnosis. Early recognition is essential to guide appropriate counseling, avoid unnecessary interventions, and facilitate timely goals-of-care discussions. As no disease-modifying therapy currently exists, management of CJD remains supportive and palliative, underscoring the importance of prompt diagnosis.
